# Altered static and dynamic functional connectivity in childhood basic-type intermittent exotropia

**DOI:** 10.1093/braincomms/fcaf358

**Published:** 2025-09-15

**Authors:** Mengdi Zhou, Qinglei Shi, Huixin Li, Mengqi Su, Haoran Zhang, Jie Hong, Xiwen Wang, Xiang Wan, Jing Fu, Zhaohui Liu

**Affiliations:** Department of Radiology, Beijing Tongren Hospital, Capital Medical University, Beijing 100730, China; School of Medicine, Chinese University of Hong Kong (Shenzhen), Shenzhen 518172, China; Shenzhen Research Institute of Big Data, Shenzhen 518172, China; Beijing Tongren Eye Center, Beijing Tongren Hospital, Capital Medical University, Beijing Key Laboratory of Ophthalmology & Visual Sciences, Beijing 100730, China; School of Computing, National University of Singapore, Singapore 119077; School of Data Science, Chinese University of Hong Kong (Shenzhen), Shenzhen 518172, China; Beijing Tongren Eye Center, Beijing Tongren Hospital, Capital Medical University, Beijing Key Laboratory of Ophthalmology & Visual Sciences, Beijing 100730, China; Department of Radiology, Beijing Tongren Hospital, Capital Medical University, Beijing 100730, China; Shenzhen Research Institute of Big Data, Shenzhen 518172, China; Beijing Tongren Eye Center, Beijing Tongren Hospital, Capital Medical University, Beijing Key Laboratory of Ophthalmology & Visual Sciences, Beijing 100730, China; Department of Radiology, Beijing Tongren Hospital, Capital Medical University, Beijing 100730, China

**Keywords:** intermittent exotropia, static functional connectivity, dynamic functional connectivity, resting-state functional magnetic resonance imaging

## Abstract

This study aimed to investigate static and dynamic functional connectivity alterations between the primary visual cortex, secondary visual cortex, higher visual cortex and oculomotor cortex in children with basic-type intermittent exotropia. A total of 44 children with basic-type intermittent exotropia and 37 healthy controls matched for sex, age and education level were included and underwent resting-state functional MRI. Both sides of Brodmann area (BA) 17, BA18, BA19 and BA8 were chosen as regions of interest. Sliding window method and *k*-means clustering analysis were employed to investigate static and dynamic functional connectivity as well as temporal metrics based on visual cortices and oculomotor cortices. Differences in functional connectivity and temporal metrics were identified and subsequently correlated with clinical characteristics using Pearson correlation analysis. Diagnostic efficacy of static and dynamic functional connectivity as well as near stereoacuity was assessed using receiver operating characteristic analysis. For static functional connectivity analysis, compared with healthy controls, children with intermittent exotropia showed decreased static functional connectivity between the right higher visual cortex (BA19) and the left oculomotor cortex (BA8), as well as between bilateral oculomotor cortices (BA8). For dynamic functional connectivity analysis, children with intermittent exotropia showed increased dynamic functional connectivity variability between the right secondary visual cortex (BA18) and the left higher visual cortex (BA19), as well as between the left higher visual cortex (BA19) and the right oculomotor cortex (BA8). In addition, the mean dwell time and fraction time in a specific state characterized by negative connectivity between visual cortices and oculomotor cortices were positively correlated with the disease duration. Receiver operating characteristic analyses demonstrated that the combination of static and dynamic functional connectivity exhibited high diagnostic performance for basic-type intermittent exotropia. Children with basic-type intermittent exotropia exhibited aberrant static and dynamic functional connectivity within the bilateral visual–oculomotor cortex pathways, which might be associated with visual perception and eye movement impairments. With the prolongation of disease duration, more time spent in a specific state might be related to aggravated eye movement disorder. The combination of static and dynamic functional connectivity provides a new perspective for exploring the neuropathological mechanisms of basic-type intermittent exotropia and offers a potential neuroimaging biomarker for diagnosis.

## Teaser

Zhou *et al*. report that children with basic-type intermittent exotropia exhibited aberrant static and dynamic functional connectivity within the bilateral visual–oculomotor cortex pathways, offering novel insights into the underlying neural mechanisms of vision and eye movement impairments. The combined static and dynamic functional connectivity may serve as a potential diagnostic biomarker.

## Graphical Abstract

**Graphical Abstract:**
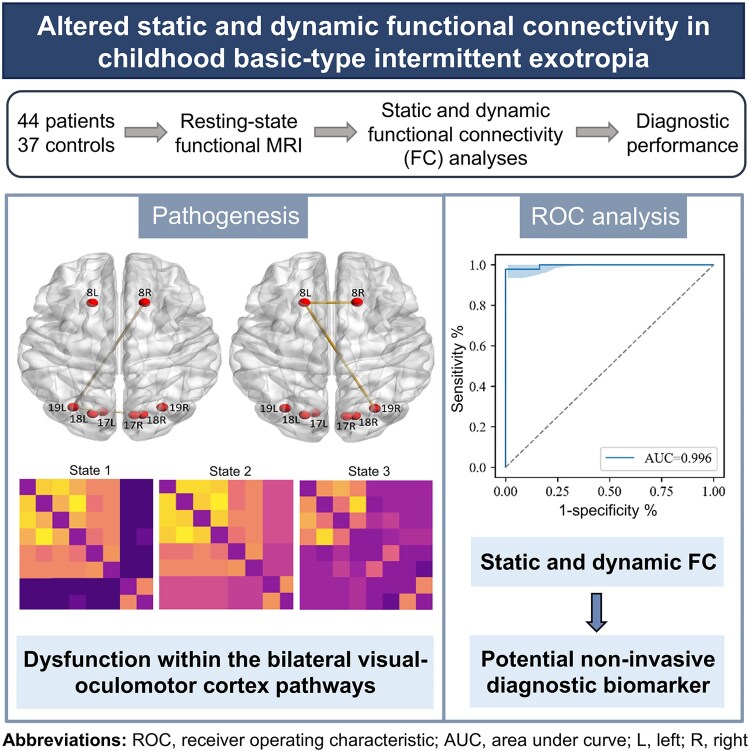

## Introduction

Intermittent exotropia (IXT) is the most common type of exotropia in childhood, with an incidence rate of nearly 1% in the United States and up to 4% in Asia.^1-3^ Among the four subtypes, basic-type IXT is the most common, accounting for 86.2%.^4^ It is characterized by dysfunction of eye movement and binocular vision.^5,6^ Cosmetic changes caused by eye deviation make children suffer from exclusion and prejudice, which gives rise to psychological problems and impaired social interaction.^7^ If left untreated, childhood-onset IXT is likely to gradually deteriorate into constant exotropia, ultimately resulting in irreversible visual function impairment.^8,9^ Although diagnosed through clinical examination, missed diagnosis frequently occurs because of the intermittent nature of IXT and the limited competency of children.^10^ The high recurrence rate of 51.1–74.1% after the extraocular muscle surgeries indicates the suboptimal surgical outcome.^11,12^ It is crucial to make an accurate early diagnosis and choose optimal treatment in the management of IXT. However, the exact pathogenesis remains unclear.

Accumulating evidence indicate that cerebral deficits are essential in the genesis of IXT.^13-18^ The primary visual cortex (V1), which receives inputs from both eyes, is the core component of binocular vision fusion.^19^ The visual information is transmitted to higher-level visual processing areas along the dorsal and ventral pathways.^20,21^ V1 [Brodmann area (BA) 17] and V2 (BA18) are components of both streams. BA19 is reputed to contain V3, V4, V5 and V6.^22^ The dorsal stream, involving BA17, BA18, the dorsal region of BA19 and a part of parietal lobe, primarily takes part in the processing of spatial position information and eye movement.^23^ The ventral stream includes BA17, BA18, the ventral region of BA19 and a part of temporal lobe and is responsible for processing colour and shape information.^24^ The dorsal and ventral processing streams converge in the oculomotor cortex (BA8), which is responsible for vergence, saccadic, smooth pursuit eye movements and ocular accommodation.^25,26^ If the visual–oculomotor cortex pathway is damaged, the binocular visual information could not be converted into appropriate eye movement signals, leading to eye position deviation.^27^

Research has shown that different subtypes of IXT have varying surgical outcomes, suggesting different underlying pathogenesis for each subtype.^28^ Two studies on brain changes of basic-type IXT contributed to comprehend the underlying neural mechanisms. Firstly, Zhang *et al*. observed functional changes in fusional vergence-related areas using task-based functional magnetic resonance imaging (task-fMRI). They suggested that reduced brain activation in the right frontal eye field, left inferior parietal lobule and cerebellum may impair fusional vergence function, whereas increased brain activation in the left middle temporal gyrus complex may compensate for this dysfunction.^29^ Unlike resting-state fMRI (rs-fMRI), task-fMRI cannot avoid potential performance confounders associated with activation paradigms.^30^ Secondly, Fei *et al*. found that children with basic-type IXT showed structural changes in visual and oculomotor areas, such as reduced grey matter density in the right frontal eye field and bilateral inferior parietal lobule, and increased cortical thickness in the right V4 area and bilateral inferior parietal lobule.^13^ However, morphological changes typically manifest later than functional abnormalities and could not accurately reflect early-stage brain alterations.^31,32^ In addition, they also observed fractional amplitude of low-frequency fluctuations and static functional connectivity (sFC) changes by rs-fMRI, such as reduced fractional amplitude of low-frequency fluctuations in the left lingual gyrus, right inferior occipital gyrus and reduced sFC between the left frontal eye field and bilateral inferior parietal lobule.^13^ Distinct from fractional amplitude of low-frequency fluctuations only characterizing local temporal coherence, FC can capture the temporal coherence of spontaneous brain activity between spatial regions.^33-35^ He *et al*. reported IXT adults had lower sFC of bilateral V1 with other visual and oculomotor areas, including the right calcarine, right superior occipital gyrus and right cuneus.^14^ Furthermore, Li *et al*. found IXT adults exhibited altered sFC within multiple resting-state networks related to binocular fusion, stereopsis, oculomotor control and emotional processes, including the default mode network, the dorsal attention network, the visual network, the sensorimotor network, the executive control network, the frontoparietal network and the auditory network.^36^ These findings indicated specific functional irregularities in visual and oculomotor areas of IXT. However, previous studies primarily focused on static intrinsic brain activity and connectivity, ignoring the important dynamic aspect over time. Moreover, confining seed-based analysis to bilateral V1 is insufficient; other key regions of the visual–oculomotor cortex pathway should also be incorporated to characterize functional interactions between visual and oculomotor areas.

The brain is a dynamic system of information interaction and functional coupling that undergoes dynamic reconfiguration at different timescales.^37^ Traditional sFC assesses the average value of the correlation between fluctuation signals, limiting its ability to reflect the dynamics and effectiveness of information interaction in the brain.^33-35^ As a powerful complement to sFC, dynamic FC (dFC) could capture time-varying continuous features on short time scales.^35^ In a dFC study of comitant exotropia, Chen *et al.* found reduced dFC between bilateral V1 and other vision and eye movement–related areas, including bilateral fusiform, bilateral lingual gyrus, left precuneus, left calcarine, left orbital medial frontal gyrus and left inferior occipital gyrus.^38^ Nevertheless, the results could not exactly reflect dFC alterations of IXT, a subtype of comitant exotropia. Integrated analysis of sFC and dFC offers superior classification efficacy and accuracy in neuroimaging disorders classification compared to using either method alone.^39-41^ This combined approach has proven to be efficient in uncovering underlying mechanisms and identifying promising neuroimaging markers for diagnosis in various neuropsychiatric disorders, such as obstructive sleep apnoea,^42^ subjective cognitive decline,^43^ and autism spectrum disorder.^44^

Therefore, this study aimed to explore sFC and dFC alterations between the primary visual cortex, secondary visual cortex, higher visual cortex and oculomotor cortex in children with basic-type IXT by using rs-fMRI and sliding window method. The correlations between aberrant FC, temporal metrics and clinical characteristics were also examined. In addition, we applied receiver operating characteristic (ROC) analysis to examine the diagnostic performance of the aberrant FC and near stereoacuity. Based on previous studies, we hypothesized that children with basic-type IXT would exhibit altered sFC and dFC within the visual–oculomotor cortex pathway and that the aberrant FC could serve as a potential biomarker for revealing the underlying neural mechanism.

## Materials and methods

### Study design and participants

This study was approved by the Ethics Committee and Institutional Review Board of Capital Medical University, Beijing Tongren Hospital (TRECKY2020-139). The authors state that all of the procedures contributing to this research comply with the ethical standards of the relevant national and institutional committees on human experimentation and with the 1975 Declaration of Helsinki, as revised in 2008. Written informed consent was obtained from all participants and their guardians. A total of 46 children with basic-type IXT (age range: 9–16 years, mean ± SD: 10.92 ± 1.54 years; 23 males) were recruited at the Beijing Tongren Hospital. In addition, 37 healthy controls (HCs) (age range: 9–16 years, mean ± SD: 11.14 ± 2.06 years; 14 males) matched for age, sex and education level were recruited at schools. All participants underwent detailed ophthalmological examinations, including best-corrected visual acuity (BCVA), fundus examination and random-dot stereogram. Near stereoacuity was measured with a random-dot stereogram (Stereo Optical Co. Inc., Chicago, IL, USA). For IXT patients, further assessments were performed, including disease duration, Newcastle Control Score (grading the ability of controlling exotropia) and prism alternate cover test (quantifying the angle of deviation).

All subjects were right-handed. The inclusion criteria for children with basic-type IXT were as follows: (i) age between 9 and 16 years, (ii) diagnosed as basic-type IXT based on history and ocular examinations and (iii) BCVA ≥ 1.0.

Participants were excluded if they had any of the following: (i) other kinds of strabismus, such as constant exotropia, esotropia or incomitant strabismus; (ii) ocular disease (e.g. amblyopia, cataract, glaucoma, optic neuritis and macular degeneration); (iii) history of treatment or eye surgery; (iv) history of brain, psychiatric or systemic disorders; (v) MR imaging contraindications (metal in cardiac pacemakers or prostheses) and (vi) low-quality MRI images.

### Data acquisition

The participants were examined using a 3.0 T MRI scanner (Siemens Healthiness, Erlangen, Germany) with a standard 64 eight-channel head coil. Foam pads were placed around the head to restrict head motion, and earplugs were used to reduce scanner noise. The rs-fMRI data were acquired using a simultaneous multi-slice echo planar imaging sequence with the following parameters: time of repetition = 1500 ms, time of echo = 30 ms, flip angle = 70°, voxel size = 2.0 × 2.0 × 2.0 mm, field of view = 220 × 220 mm, matrix = 110 × 110, slice thickness = 2 mm and 340 time points. The scanning time was 8 min and 45 s. Three - dimensional (3D) T1-weighted anatomical images were obtained using a 3D magnetization prepared rapid acquisition gradient echo sequence: time of repetition = 2000ms, time of echo = 2.25 ms, inversion time = 900 ms, flip angle = 8°, voxel size = 1 × 1 × 1 mm, field of view = 256 × 256 mm, matrix = 256 × 256, slice thickness = 1.0 mm and 192 slices. The scanning time was 4 min and 6 s.

### Resting-state functional MRI preprocessing

The processing of rs-fMRI data was carried out using the DPABI (version 7.0; http://rfmri.org/dpabi/) and SPM12 toolboxes in MATLAB platform (MathWorks, Natick, MA, USA).^45^ First, raw images were converted from DICOM to NIFTI format, and the initial 10 time points were removed to stabilize signals. The remaining images underwent slice timing and head motion correction. The data were then normalized to Montreal Neurological Institute standard space, with voxel sizes resampled to 3 × 3 × 3 mm. Spatial smoothing was conducted with a Gaussian kernel of 6 mm full-width at half-maximum. The linear trend of the time series was removed, and temporal band-pass filtering (0.01–0.08 Hz) was applied. Friston-24 motion parameters, whole brain, white matter and cerebrospinal fluid signals were regressed out as the covariates.^46^ Participant data with maximum head translation (rotation) motion > 4 mm (4°) were discarded.

### Definition of the region of interest

Both sides of BA17 (primary visual cortex), BA18 (secondary visual cortex), BA19 (higher visual cortex) and BA8 (oculomotor cortex) of Brodmann atlas were chosen as regions of interest (ROIs). Using the MarsBar software (https://sourceforge.net/projects/marsbar/), eight spherical ROIs with a 5 mm radius were extracted in every group.

### sFC and dFC analyses

The sFC and dFC analyses were conducted using the DynamicBC software (V2.2, http://restfmri.net/forum/DynamicBC). For sFC analysis, an 8 × 8 sFC matrix was generated for each subject by computing pairwise Pearson correlation coefficients between the time series of eight ROIs across the entire fMRI scan duration (330 time points). The dFC analysis was analysed using a sliding window approach. Each window contained 50 time points with a step size of 20 time points (60% overlap), resulting in 15 overlapping windows for each subject. For each sliding window, Pearson correlation coefficients between all ROI pairs were computed, yielding 15 sliding window correlation maps for each subject. To capture the temporal fluctuations in FC, we calculated the variance of the dFC maps across the 15 sliding windows, resulting in an 8 × 8 dFC variability matrix for each subject.

### Clustering analysis

The *k*-means clustering algorithm with the squared Euclidean distance was applied to identify reoccurring FC patterns (states).^47^ In this study, we determined the optimal number of clusters *k* based on the averaged results of three standard cluster validity indices, including the Silhouette score, Calinski–Harabasz index and Davies–Bouldin index. Clustering numbers ranging from 2 to 6 were selected to represent the cluster states. For each value of *k*, the clustering algorithm was repeated 500 times in MATLAB to reduce the bias of random initialization of centroid position. Three (*k* = 3) was determined as the optimal clustering number, namely, States 1, 2 and 3. The medians of all state-assigned FC matrices across time were used to obtain the final cluster centroids.

Several temporal metrics were computed, including (i) the fraction time, representing the percentage of time spent in each of the three states out of the total time; (ii) mean dwell time, indicating the average duration (in consecutive time windows) a subject remained in a specific state before transitioning to another, and (iii) number of transitions, indicating the number of times a subject switched between different states.

### Statistical analysis

The SPSS 26.0 software package (SPSS, Inc., Chicago, IL, USA) was used for statistical analysis. The normality of demographic and clinical data was assessed using the Shapiro–Wilk test. A two-sample *t*-test was used for normally distributed data. Categorical variables were conveyed as counts, and the comparison of genders was conducted using the chi-square test. The significance threshold was set at *P* < 0.05.

A two-sample *t*-test was performed to analyse sFC and dFC variability differences between the two groups while controlling for age, sex and education level as nuisance covariance. The statistical threshold was set at a voxel level of *P* < 0.001 and a cluster level of *P* < 0.05, Gaussian random field corrected. Group differences in the fraction time, mean dwell time and number of transitions were examined using a two-sample *t*-test, while controlling for age, sex and education level as nuisance covariance (*P* < 0.05, false discovery rate corrected). The degrees of freedom for the two-sample *t*-test were 79. Pearson correlation analysis was performed to correlate altered FC and temporal metrics with clinical characteristics such as disease duration, near deviation, distance deviation, Newcastle Control Score and near stereoacuity, while controlling for age, sex and education level.

The diagnostic performance of sFC, dFC variability and near stereoacuity was evaluated using ROC analysis. As for aberrant FC, we assessed the diagnostic performance of (i) sFC between the right BA19 and the left BA8, sFC between bilateral BA8 and the combined sFC of these two connections; (ii) dFC variability between the left BA19 and the right BA8, dFC variability between the left BA19 and the right BA18, and the combined dFC variability of these two connections; and (iii) the combination of altered sFC and dFC variability. The area under the curve (AUC) was used to measure the overall performance of the diagnostic tests, with values ranging from 0 to 1.

## Results

### Demographics and clinical characteristics

Two children with IXT were excluded from the analysis because of excessive head movement. The remaining 44 children with basic-type IXT (age range: 9–16 years, mean ± SD: 10.95 ± 1.57 years; 22 males) and 37 HCs (age range: 9–16 years, mean ± SD: 11.14 ± 2.06 years; 14 males) matched for sex, age and education level were included in this study.

Clinical characteristics of children with IXT and HCs were summarized in Table 1. No significant differences were found between children with IXT and HCs in age, sex, handedness, education level or BCVA. There was a significant difference in near stereoacuity between the two groups. Among children with IXT, the average disease duration was 3.18 ± 2.44 years (ranging from 0.5 to 9), the mean angle of exodeviation was 49.55 ± 20.65 prism dioptres at near (ranging from 15 to 110) and 44.66 ± 21.22 prism dioptres at distance (ranging from 15 to 100) and the mean near stereoacuity was 2.58 ± 0.55 log arcsec. In the HC group, the mean near stereoacuity was 1.95 ± 0.28 log arcsec.

**Table 1:** Demographic data and clinical features of children with IXT and HC

	IXT (*n* = 44)	HC (*n* = 37)	*P*-value
Female, *n* (%)	22 (50)	23 (62.2)	0.273^a^
Age, years	10.95 (1.57)	11.14 (2.06)	0.656^b^
Handedness (right), *n* (%)	44 (100)	37 (100)	
Education, years	5.30 (1.64)	5.32 (2.16)	0.946^b^
BCVA of right eye	1.00 (0.06)	1.01 (0.06)	0.774^b^
BCVA of left eye	1.00 (0.08)	1.02 (0.09)	0.453^b^
Duration, years	3.18 (2.44)	N/A	N/A
Near deviation, PD	49.55 (20.65)	N/A	N/A
Distance deviation, PD	44.66 (21.22)	N/A	N/A
Newcastle Control Score	4.82 (2.08)	N/A	N/A
Near stereoacuity, log arcsec	2.58 (0.55)	1.95 (0.28)	0.001^c^

Data are presented as mean (SD) for continuous variables and number (%) for categorical variables. IXT, intermittent exotropia; HC, healthy control; N/A, not applicable; BCVA, best-corrected visual acuity; PD, prism dioptres.

^a^ *P*-value was assessed by the chi-squared test.

^b^ *P*-value was assessed by the two-sample *t*-test.

^c^ *P* < 0.05.

### Differences in sFC and dFC between groups

**Figure 1:**
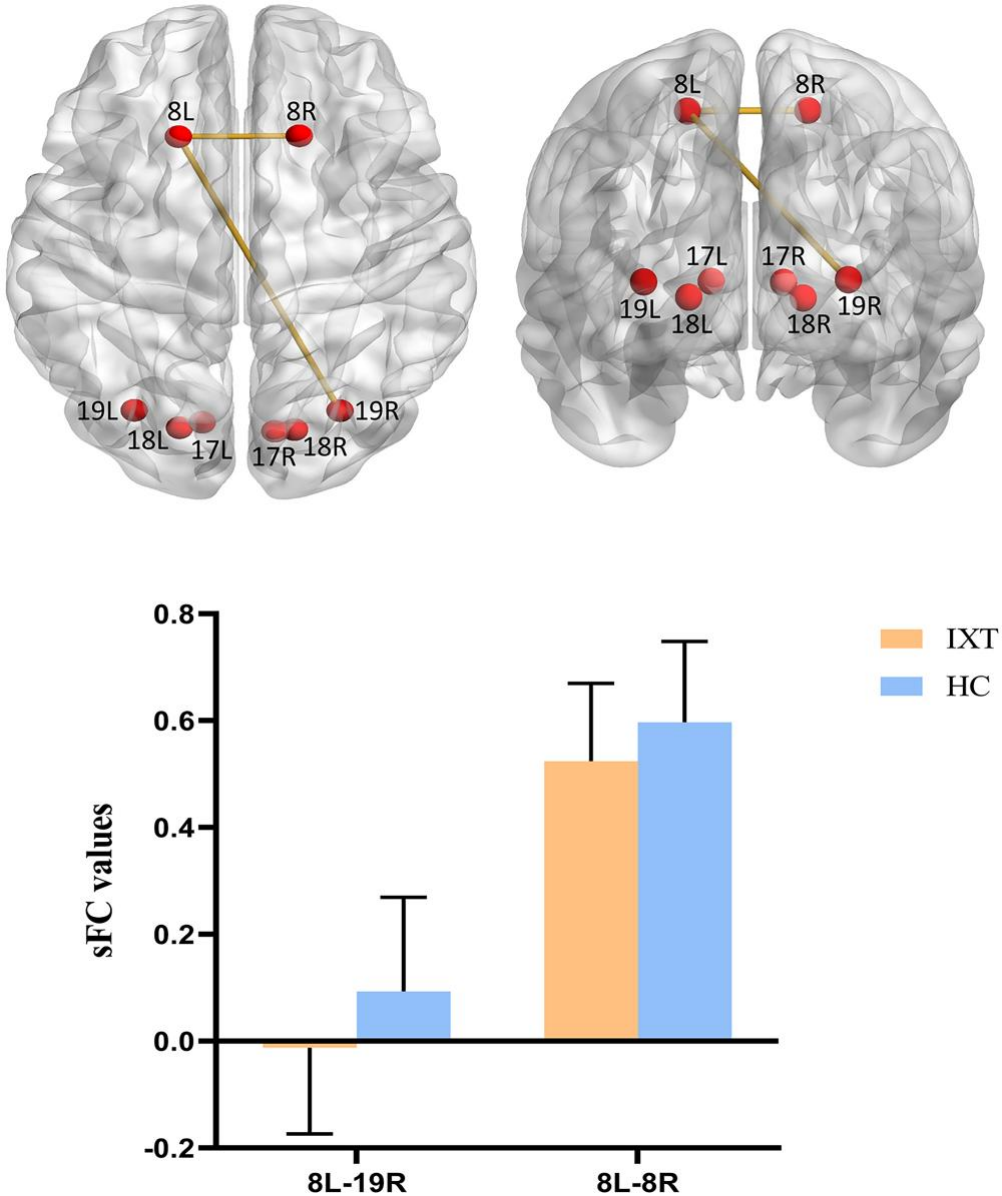
**Group differences in sFC between the IXT and HC groups.** Compared with the HC group, the IXT group showed decreased sFC between the right BA19 and left BA8 as well as between the bilateral BA8. The red balls represent the seed ROIs, and the connecting columns between the ROIs indicate significant differences in sFC between the two groups. Data are presented as mean ± SD (IXT, *n* = 44; HC, *n* = 37), with statistical analysis performed using a two-sample *t*-test, Gaussian random field correction with a threshold of voxel level *P* < 0.001, and cluster level *P* < 0.05. sFC, static functional connectivity; IXT, intermittent exotropia; HC, healthy control; ROI, region of interest; BA, Brodmann area;17L/R, left/right Brodmann area 17; 18L/R, left/right Brodmann area 18; 19L/R, left/right Brodmann area 19; 8L/R, left/right Brodmann area 8.

**Figure 2:**
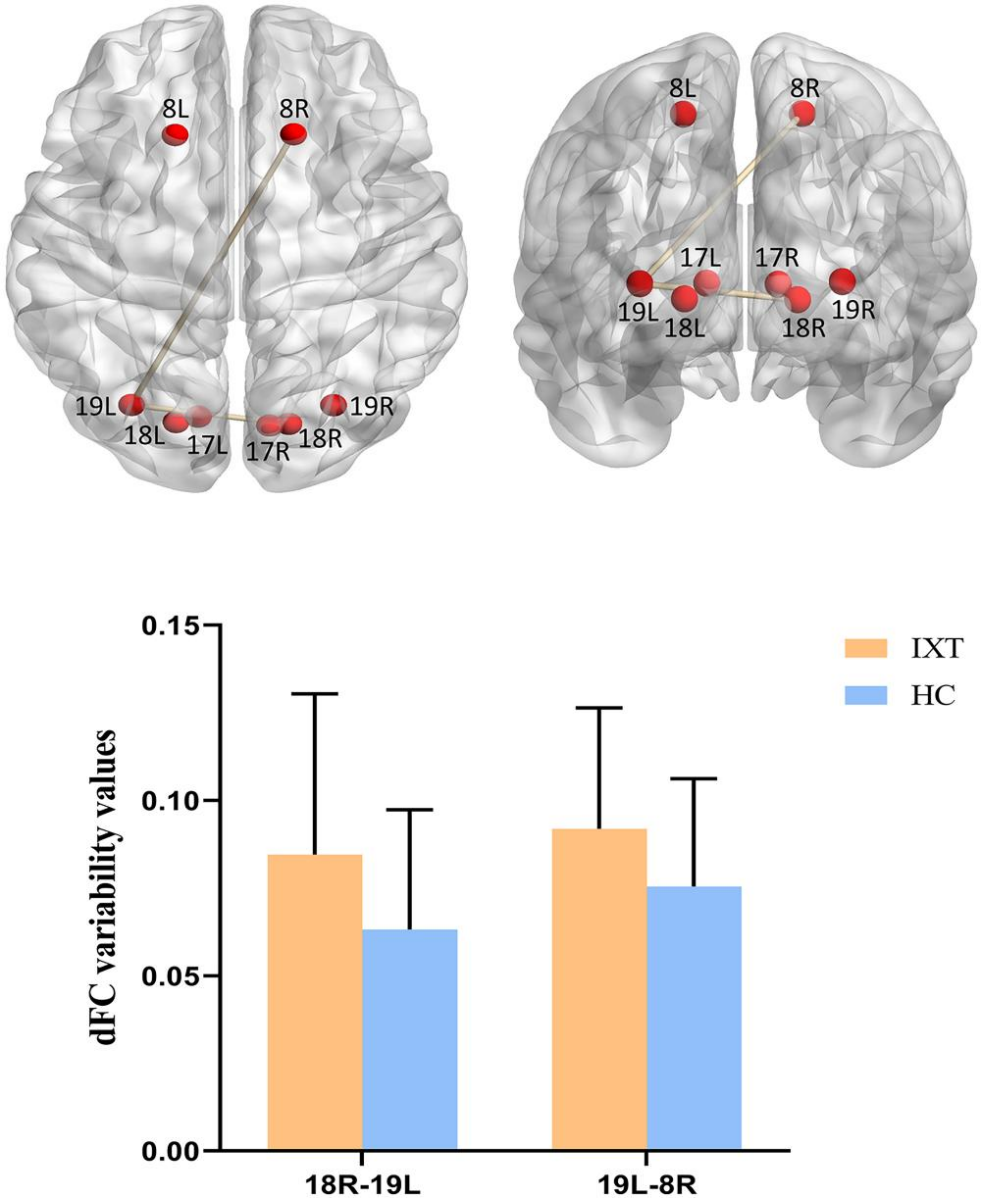
**Group differences in dFC variability between the IXT and HC groups.** Compared with the HC group, the IXT group showed increased dFC variability between the right BA18 and left BA19 as well as between the left BA19 and right BA8. The red balls represent the seed ROIs, and the connecting columns between the ROIs indicate significant differences in dFC variability between the two groups. Data are presented as mean ± SD (IXT, *n* = 44; HC, *n* = 37), with statistical analysis performed using a two-sample *t*-test, Gaussian random field correction with a threshold of voxel level *P* < 0.001, and cluster level *P* < 0.05. dFC, dynamic functional connectivity; IXT, intermittent exotropia; HC, healthy control; ROI, region of interest; BA, Brodmann area; 17L/R, left/right Brodmann area 17; 18L/R, left/right Brodmann area 18; 19L/R, left/right Brodmann area 19; 8L/R, left/right Brodmann area 8.

For sFC analysis, compared with HCs, children with IXT showed decreased sFC between the right higher visual cortex (BA19) and the left oculomotor cortex (BA8) and decreased sFC between bilateral oculomotor cortices (BA8) (Fig. 1). For dFC analysis, children with IXT showed increased dFC variability between the right secondary visual cortex (BA18) and the left higher visual cortex (BA19) and increased dFC variability between the left higher visual cortex (BA19) and the right oculomotor cortex (BA8) (Fig. 2).

### dFC state analysis

**Figure 3:**
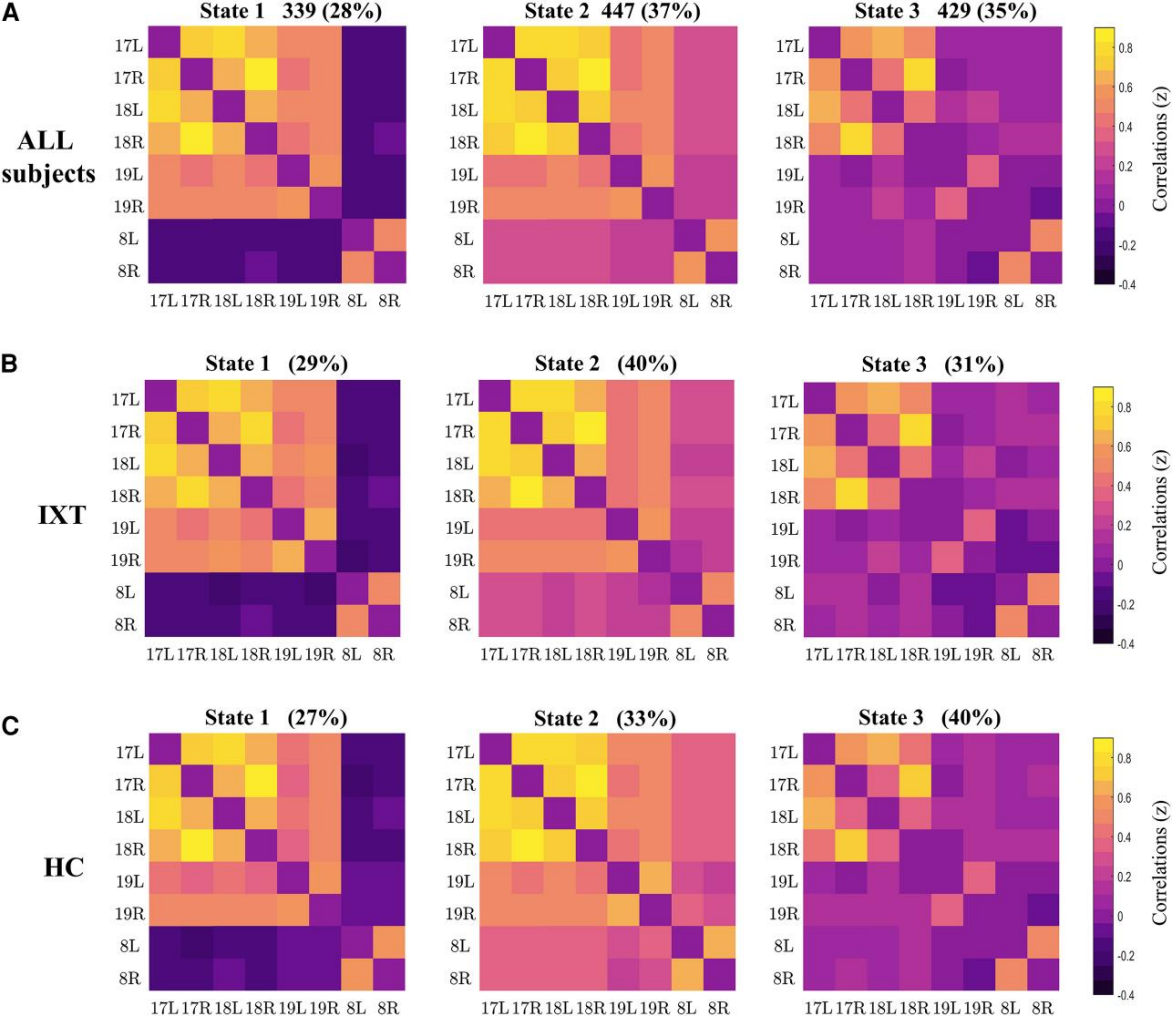
**Cluster centroids of three states identified by the *k-*means clustering method for all subjects, the IXT group and the HC group.** (**A**) Cluster centroids of three states for all subjects (*n* = 81). The total number of occurrences and percentage of total occurrences were listed above each state [State 1: 339 (28%), State 2: 447 (37%) and State 3: 429 (35%)]. (**B**) Cluster centroids of three states for the IXT group (*n* = 44). The percentages of total occurrences were listed above each state (State 1: 29%, State 2: 40% and State 3: 31%). (**C**) Cluster centroids of three states for the HC group (*n* = 37). The percentages of total occurrences were listed above each state (State 1: 27%, State 2: 33% and State 3: 40%). IXT, intermittent exotropia; HC, healthy control; 17L/R, left/right Brodmann area 17; 18L/R, left/right Brodmann area 18; 19L/R, left/right Brodmann area 19; 8L/R, left/right Brodmann area 8.

Using the *k*-means clustering method, three separate states were determined from all dFC windows. The group-specific cluster centroids of children with IXT and HCs in each state had similar connectivity properties to the cluster centroids obtained across all subjects (Fig. 3). The percentages of total occurrences of these three states in all subjects were different, with State 1 (28%), State 2 (37%) and State 3 (35%) (Fig. 3A). For children with IXT, the percentages of total occurrences of States 1, 2 and 3 was 29, 40 and 31% (Fig. 3B). For HCs, the percentages of total occurrences of States 1, 2 and 3 was 27, 33 and 40% (Fig. 3C). State 1 exhibited relatively strong positive connectivity between bilateral BA17, between bilateral BA18, between ipsilateral BA17 and BA18 and between contralateral BA17 and BA18 and exhibited negative connectivity between visual cortices and oculomotor cortices, such as between bilateral BA8 and bilateral BA17, BA18 and BA19. State 2 exhibited large-scale positive connectivity, showing relatively strong positive connectivity between bilateral BA17, between bilateral BA18, between ipsilateral BA17 and BA18 and between contralateral BA17 and BA18. State 3 had sparse connectivity and showed relatively strong positive connectivity between right BA17 and right BA18 and between left BA17 and left BA18. However, there were no significant differences in the mean number of transitions, as well as the fraction time and mean dwell time in each state between the two groups (*P* > 0.05) (Supplementary Table 1 and Fig. 1).

### Correlation to disease duration

**Figure 4:**
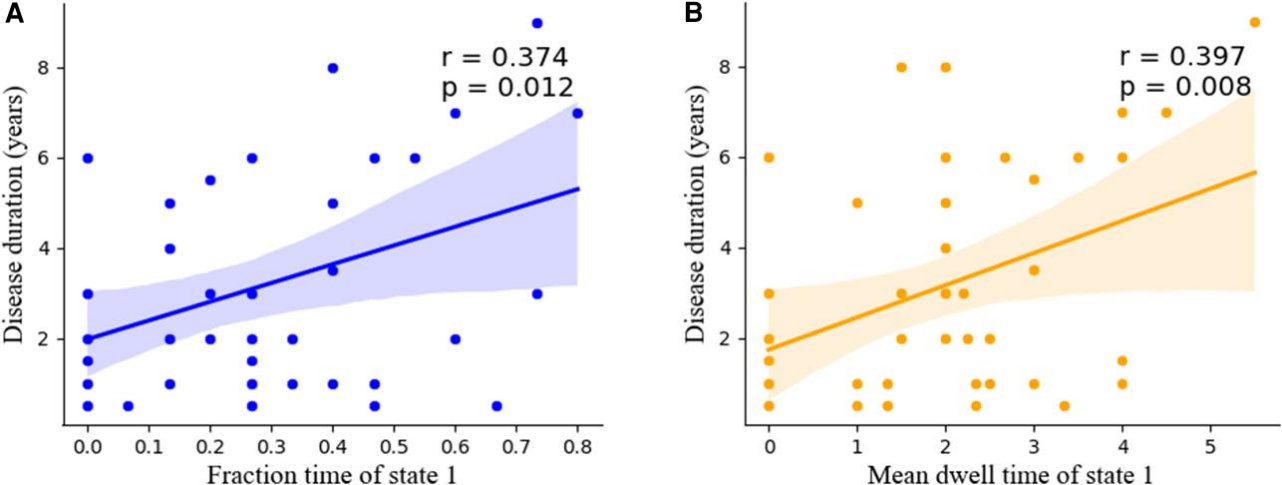
**Correlations between the temporal metrics and disease duration in the IXT group.** (**A**) The fraction time of State 1 was positively associated with the disease duration in the IXT group (IXT, *n* = 44, using Pearson correlation analysis, *r* = 0.374, *P* = 0.012). The blue points represent the distribution of data in the IXT group. (**B**) The mean dwell time of State 1 was positively associated with the disease duration in the IXT group (IXT, *n* = 44, using Pearson correlation analysis, *r* = 0.397, *P* = 0.008). The orange points represent the distribution of data in the IXT group. IXT, intermittent exotropia.

The fraction time of State 1 was positively associated with the disease duration of children with IXT (*r* = 0.374, *P* = 0.012) (Fig. 4A), suggesting that as the prolongation of disease duration, a greater percentage of time spent in State 1. Also, the mean dwell time of State 1 was positively associated with the disease duration of children with IXT (*r* = 0.397, *P* = 0.008) (Fig. 4B), indicating that with the prolongation of disease duration, the dwell time in State 1 extended. No correlation was found between clinical characteristics and altered sFC and dFC as well as the mean number of transitions.

### Diagnostic performance of aberrant FC and near stereoacuity

**Figure 5:**
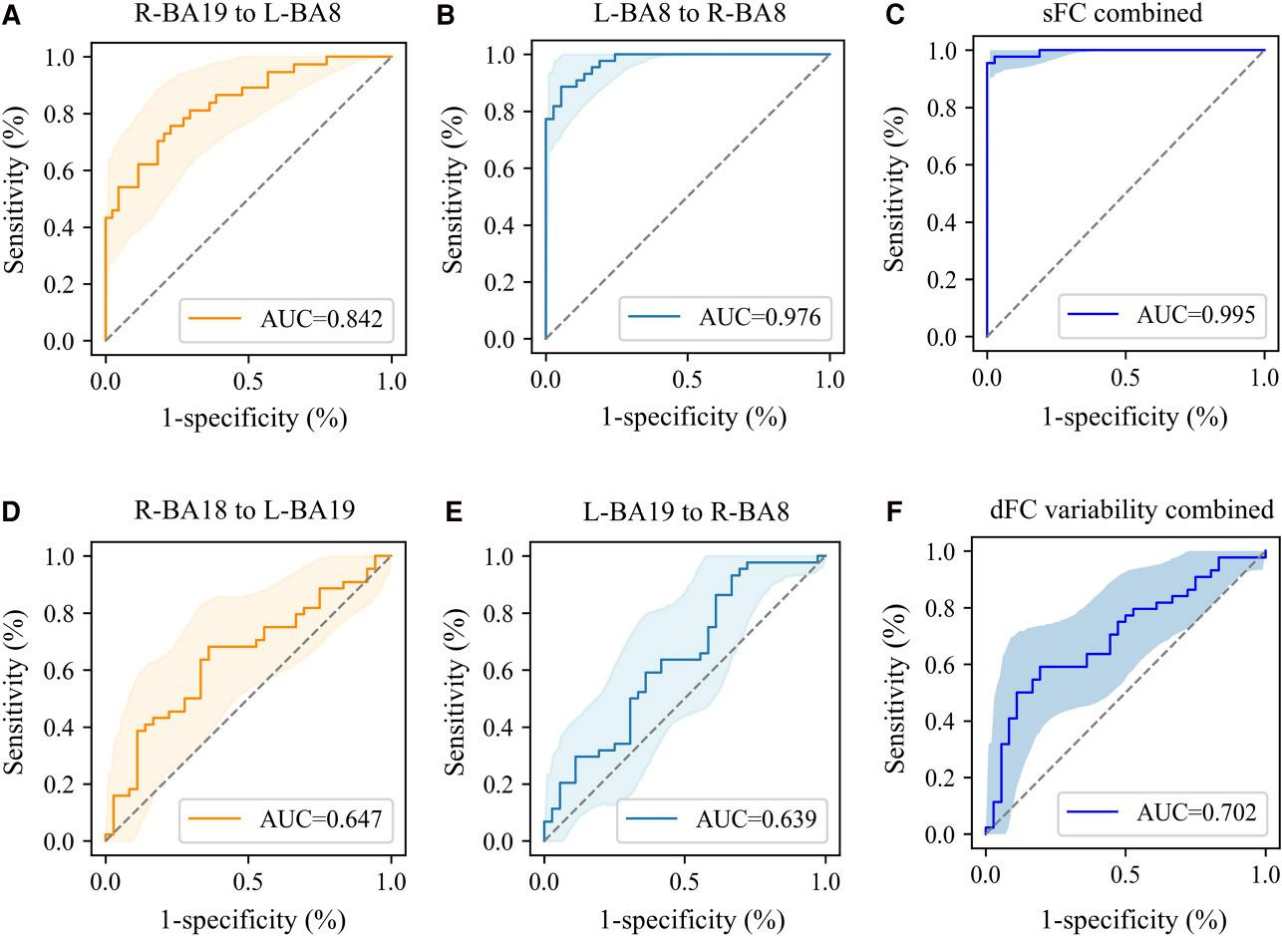
**ROC analysis based on sFC and dFC variability.** (**A–C**) ROC curves illustrating the classification of IXT group and HC group based on sFC. The AUC values of sFC between the right BA19 and left BA8, sFC between the left BA8 and right BA8 and the combined sFC of these connections were 0.842, 0.976 and 0.995, respectively (IXT, *n* = 44; HC, *n* = 37). (**D–F**) ROC curves presenting the classification of IXT group and HC group based on dFC variability. The AUC values of the dFC variability between the right BA18 and left BA19, dFC variability between the left BA19 and right BA8 and the combined dFC variability of these connections were 0.647, 0.639 and 0.702, respectively (IXT, *n* = 44; HC, *n* = 37). ROC, receiver operating characteristic; sFC, static functional connectivity; dFC, dynamic functional connectivity; IXT, intermittent exotropia; HC, healthy control; BA, Brodmann area; L, left; R, right; AUC, area under the curve.

**Figure 6:**
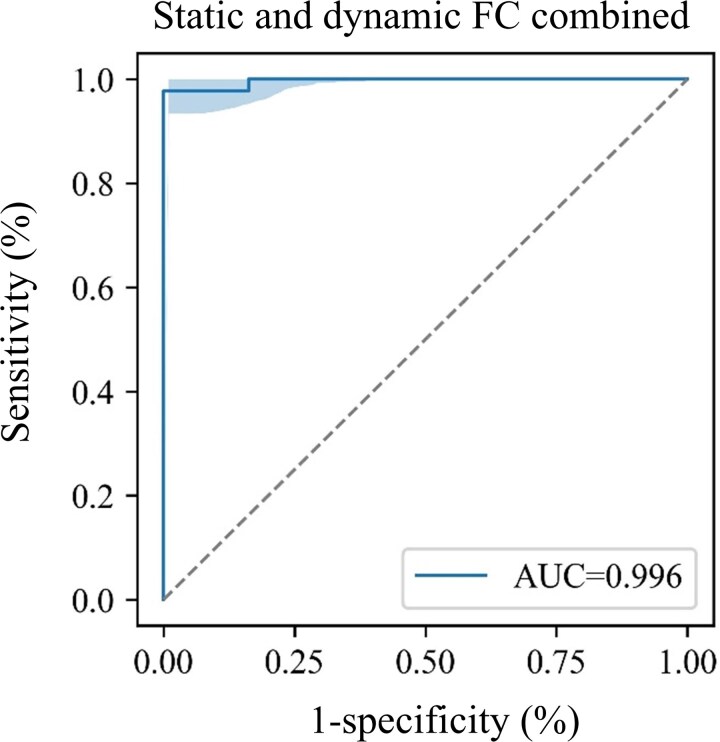
**ROC analysis based on the combination of sFC and dFC variability.** The result demonstrated that integrating dFC variability with altered sFC further improved diagnostic performance. The AUC value of the combination of sFC and dFC variability was 0.996 (IXT, *n* = 44; HC, *n* = 37). ROC, receiver operating characteristic; sFC, static functional connectivity; dFC, dynamic functional connectivity; AUC, area under the curve.

The diagnostic efficacy of aberrant FC was shown in Table 2 and Figs 5 and 6. The ROC curves showed that sFC demonstrated higher diagnostic power in discriminating children with IXT from HCs compared to dFC variability. Notably, the sFC between bilateral BA8 achieved a high diagnostic performance, with an AUC of 0.976, sensitivity of 0.886 and specificity of 0.945 (Fig. 5B and Table 2). When combined with the sFC between right BA19 and left BA8, the diagnostic performance further improved, with the AUC increasing by 1.9%, sensitivity by 6.8% and specificity by 5.5% (Fig. 5C and Table 2). Moreover, integrating altered sFC and dFC variability yielded additional improvements, with the AUC increasing by 0.1%, sensitivity by 2.3% and negative predictive value by 2.6% (Fig. 6 and Table 2).

**Table 2:** Diagnostic performance of sFC and dFC variability

Metrics	AUC (95% CI)	Sensitivity (95% CI)	Specificity (95% CI)	NPV (95% CI)	PPV (95% CI)
sFC					
R-BA19 to L-BA8	0.842 (0.666–0.855)	0.756 (0.608–0.875)	0.772 (0.638–0.892)	0.791 (0.659–0.902)	0.737 (0.589–0.875)
L-BA8 to R-BA8	0.976 (0.851–0.965)	0.886 (0.791–0.975)	0.945 (0.868–1.000)	0.875 (0.764–0.973)	0.951 (0.878–1.000)
Static combined	0.995 (0.941–1.000)	0.954 (0.886–1.000)	1.000 (1.000–1.000)	0.948 (0.875–1.000)	1.000 (1.000–1.000)
dFC variability					
R-BA18 to L-BA19	0.647 (0.564–0.772)	0.681 (0.536–0.818)	0.648 (0.485–0.781)	0.632 (0.471–0.781)	0.698 (0.545–0.837)
L-BA19 to R-BA8	0.639 (0.544–0.719)	0.931 (0.850–1.000)	0.324 (0.173–0.486)	0.800 (0.571–1.000)	0.621 (0.507–0.728)
Dynamic combined	0.702 (0.588–0.780)	0.545 (0.394–0.690)	0.838 (0.714–0.956)	0.608 (0.471–0.745)	0.800 (0.652–0.933)
All combined	0.996 (0.963–1.000)	0.977 (0.927–1.000)	1.000 (1.000–1.000)	0.974 (0.912–1.000)	1.00 (1.000–1.000)

sFC, static functional connectivity; dFC, dynamic functional connectivity; BA, Brodmann area; L, left; R, right; AUC, area under the curve; NPV, negative predictive value; PPV, positive predictive value; CI, confidence interval.

The diagnostic efficacy of near stereoacuity was shown in Supplementary Table 2 and Fig. 2. The ROC curve revealed that the diagnostic accuracy of near stereoacuity, with an AUC of 0.731, was lower than that of the combination of sFC and dFC variability, with an AUC of 0.996.

## Discussion

The current study employed a combined static and dynamic approach to investigate aberrant FC patterns between the primary visual cortex, secondary visual cortex, higher visual cortex and oculomotor cortex in children with basic-type IXT. Our main findings are as follows: (i) children with IXT showed decreased sFC and increased dFC variability between the right secondary visual cortex and the left higher visual cortex, between bilateral higher visual cortices and oculomotor cortices and between bilateral oculomotor cortices; (ii) the fraction time and mean dwell time in a specific state characterized by negative connectivity between visual cortices and oculomotor cortices were positively associated with the disease duration; and (iii) the ROC analyses revealed the combination of sFC and dFC variability exhibited high diagnostic performance for basic-type IXT. Overall, these results demonstrated that children with IXT exhibited both sFC and dFC abnormalities within the bilateral visual–oculomotor cortex pathways, which may help explain the underlying neural mechanisms of visual perception and eye movement deficits. Furthermore, the combination of sFC and dFC variability can serve as a potential neuroimaging biomarker for discriminating children with basic-type IXT from HCs. These findings further support the hypothesis that dysfunction within the visual–oculomotor pathway could contribute to the pathogenesis of basic-type IXT.

Children with IXT showed increased dFC variability between the right secondary visual cortex (BA18) and the left higher visual cortex (BA19). Increased temporal variability might reflect inappropriate processing of information.^48^ This finding aligns with previous investigations that have revealed reduced cortical activity in BA18 and BA19 of patients with IXT.^13,15,17^ BA18 and BA19 receive binocular fusion signals from V1 and are primarily involved in stereopsis formation.^49,50^ The brain uses binocular disparity to extract depth information from 2D retinal images to form stereoscopic vision.^30^ Therefore, the increased dFC variability between the right secondary visual cortex and the left higher visual cortex might indicate an inability to maintain an efficient stereoscopic information processing, which possibly results in the failure to transform binocular fusion signals into normal stereoscopic vision.

We also observed that aberrant sFC and dFC variability between bilateral higher visual cortices and oculomotor cortices and between bilateral oculomotor cortices in children with IXT, such as decreased sFC of right BA19 and left BA8, increased dFC variability of left BA19 and right BA8, and decreased sFC of bilateral BA8. In line with our results, previous studies also reported decreased grey matter density and reduced brain activation in the right frontal eye field of children with basic-type IXT,^13,29^ indicating structural and functional abnormalities of BA8. Abnormal sFC and dFC variability between bilateral higher visual cortices and oculomotor cortices and between bilateral oculomotor cortices might suggest that the stereoscopic vision information from higher visual cortices could not be converted into appropriate eye movement information in bilateral oculomotor cortices, leading to eye movement disorder. In patients with IXT, although ultrastructural changes of extraocular muscles like degeneration and fibrosis have been observed with light microscopy and electron microscopy,^27^ abnormal neuronal activity within the oculomotor system plays a crucial role in eye movement dysfunction.

ROC analyses revealed that the combination of sFC and dFC exhibited superior diagnostic efficacy compared to near stereoacuity. sFC is concerned with the ‘strength’ of FC, and dFC is concerned with the instability of FC.^51^ In our study, dFC analysis not only provided additional information distinct from sFC but also added complementary diagnostic value to sFC in classification performance. By combining sFC and dFC findings, we identified functional impairments within the bilateral visual–oculomotor cortex pathways in children with basic-type IXT. This provided a neural basis for understanding the clinical symptom of alternating intermittent squinting outwards in both eyes, commonly observed in IXT patients.

We identified three stable and repetitive dFC states in all subjects. However, there were no significant differences in the mean number of transitions as well as the fraction time and mean dwell time in each state between the two groups. This may be due to the mild condition of children with IXT included in the study. The fraction time and mean dwell time of State 1 was positively associated with the disease duration of children with IXT. That is, as the disease progresses, children with IXT spent more time in State 1. State 1 was characterized by negative connectivity between visual cortices and oculomotor cortices, which indicated that the ability to process eye movement information was impaired. This finding helped explain that with the prolongation of disease duration, the impaired oculomotor control in patients with IXT was aggravated.

There are several limitations in this study. Firstly, this was a small sample study, and the results should be further verified in future with a larger sample. Secondly, as a cross-sectional study, it is incapable of observing the specific temporal relationship between brain FC alterations and disease stage, which will be investigated in future research. Finally, the selected seed regions focused on the key brain regions related to vision and eye movement. A more comprehensive range of network interaction models would be explored in the future.

## Conclusions

The present study observed aberrant sFC and dFC variability between the right secondary visual cortex and the left higher visual cortex, between bilateral higher visual cortices and oculomotor cortices and between bilateral oculomotor cortices in children with basic-type IXT, which might be associated with dysfunction in visual perception and oculomotor control. With the prolongation of disease duration, more time spent in a specific state might be related to aggravated oculomotor disorder. Notably, the ROC analyses demonstrated that the combination of sFC and dFC could serve as a potential neuroimaging biomarker for diagnosing basic-type IXT. Overall, these findings highlighted both sFC and dFC abnormalities within the bilateral visual–oculomotor cortex pathways in children with IXT, offering novel insights into the neural mechanisms underlying this condition.

## Supplementary Material

fcaf358_Supplementary_Data

## Data Availability

The data that support the findings of this study are available on request from the corresponding author. The code and instructions used in this study are publicly available at https://anonymous.4open.science/r/IXT-4F75/.
