# Supplementary material for: Altered static and dynamic functional connectivity in childhood basic-type intermittent exotropia
Source: Brain Commun. 2025 Sep 15;7(5):fcaf358. doi: 10.1093/braincomms/fcaf358 (PMC12485606; doi:10.1093/braincomms/fcaf358)
Supplement: fcaf358_Supplementary_Data [file fcaf358_supplementary_data.docx]

Supplementary Figure 1: Comparison of temporal metrics of dFC states between the IXT and HC groups. (A) Comparison of the fraction time spent in each state, (B) Comparison of the mean number of transitions, and (C) Comparison of the mean dwell time in each state. Data are presented as mean ± SD (IXT, n=44; HC, n=37), with statistical analysis performed using the two-sample t-test, false discovery rate corrected. The orange bars represent the IXT group, while the blue bars represent the HC group. There were no significant differences in the mean number of transitions, as well as the fraction time and mean dwell time in each state between the two groups (P > 0.05). dFC, dynamic functional connectivity; IXT, intermittent exotropia; HC, healthy control. **
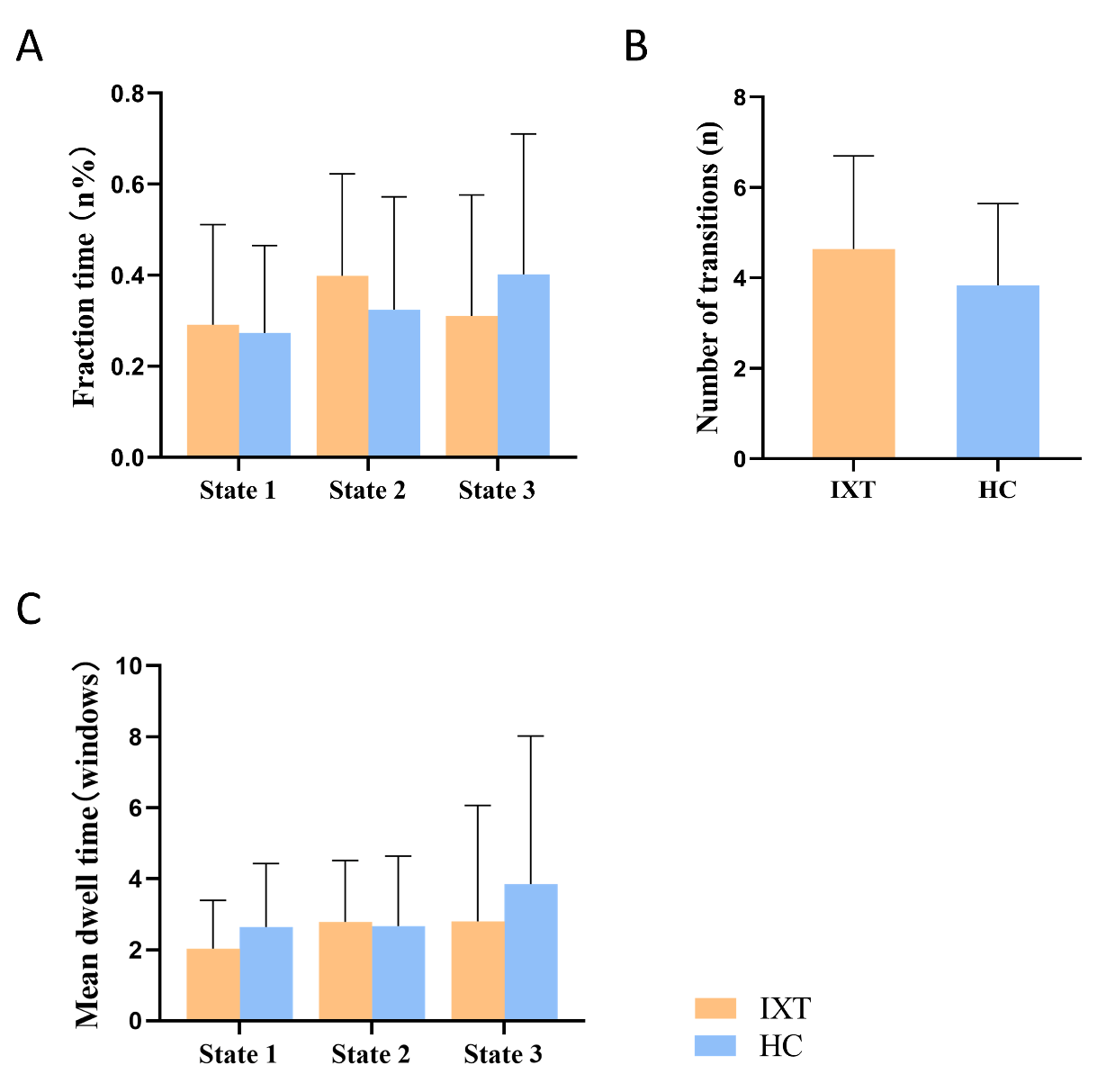
**

Supplementary Figure 2: **ROC analysis based on the near stereoacuity.** The ROC curve presenting the classification of IXT group and HC group based on near stereoacuity, with an AUC of 0.731 (IXT, n=44; HC, n=37) . ROC, receiver operating characteristic; IXT, intermittent exotropia; HC, healthy control; AUC, area under the curve.

**
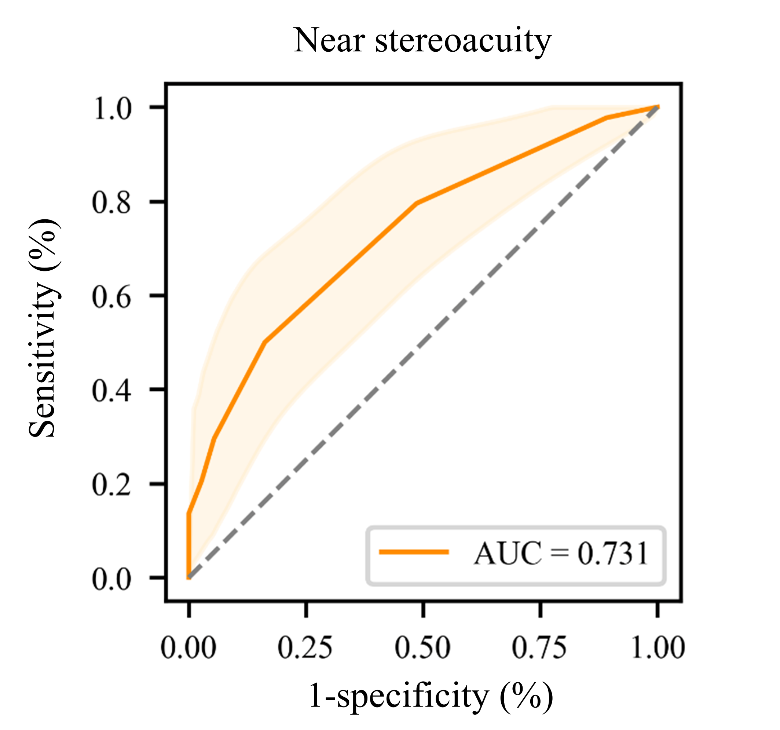
**

Supplementary **Table 1** **| Comparison of the temporal** **metrics of dFC states between the IXT and HC groups**

| **Metrics** | **IXT (n=44)** | **HC (n=37)** | ***P*-value** |
| --- | --- | --- | --- |
| mean number of transitions (n) | 4.64 (2.06) | 3.84 (1.80) | 0.070 |
| **State 1** |  |  |  |
| fraction time (n %) | 0.29 (0.22) | 0.27 (0.19) | 0.714 |
| mean dwell time (windows) | 2.03 (1.36) | 2.64 (1.78) | 0.082 |
| **State 2** |  |  |  |
| fraction time (n %) | 0.40 (0.22) | 0.32 (0.24) | 0.162 |
| mean dwell time (windows) | 2.78 (1.74) | 2.66 (1.98) | 0.779 |
| **State 3** |  |  |  |
| fraction time (n %) | 0.31 (0.27) | 0.40 (0.31) | 0.157 |
| mean dwell time (windows) | 2.79 (3.28) | 3.85 (4.17) | 0.205 |

Data are presented as median (SD). dFC, dynamic functional connectivity; IXT, intermittent exotropia; HC, healthy control.

Supplementary **Table 2** **| Diagnostic performance of near stereoacuity**

| **Metric** | **AUC**  **[95% CI]** | **Sensitivity**  **[95% CI]** | **Specificity**  **[95% CI]** | **NPV**  **[95% CI]** | **PPV**  **[95% CI]** |
| --- | --- | --- | --- | --- | --- |
| near stereoacuity | 0.731  [0.621, 0.823] | 0.500  [0.346, 0.654] | 0.838  [0.680, 0.938] | 0.786  [0.625, 0.890] | 0.585  [0.504, 0.662] |

AUC, area under the curve; NPV, negative predictive value; PPV, positive predictive value; CI, confidence interval.
